# Assessment of nutrition knowledge and associated factors among secondary school students in Haramaya district, Oromia region, eastern Ethiopia: implications for health education

**DOI:** 10.3389/fpubh.2024.1398236

**Published:** 2024-06-19

**Authors:** Alo Edin, Kedir Jemal, Ibsa Abdusemed Ahmed, Berhe Gebremichael, Abdulmalik Abdela Bushra, Melake Demena, Merian Abdirkadir

**Affiliations:** ^1^Department of Epidemiology, School of Public Health, Institute of Health, Bule Hora University, Bule Hora, Ethiopia; ^2^School of Public Health, College of Health and Medical Sciences, Haramaya University, Harar, Ethiopia; ^3^Department of Public Health, Institute of Health, Bule Hora University, Bule Hora, Ethiopia

**Keywords:** student, knowledge, nutrition, secondary school, Ethiopia

## Abstract

**Background:**

Nutrition knowledge stands as a cornerstone in facilitating informed dietary choices, thereby profoundly impacting overall health and lifestyle outcomes. Malnutrition often correlates with deficient nutritional knowledge, highlighting the critical need for comprehensive understanding in this domain. While Ethiopia has seen considerable research on nutritional status and associated factors, there remains a paucity of studies specifically addressing nutrition knowledge among secondary school students, particularly within the Haramaya District. Therefore, this study aimed to meticulously assess nutrition knowledge and its determinants among secondary school students in Eastern Ethiopia.

**Methods:**

Employing an institutional-based cross-sectional design, we carefully selected 417 students from secondary schools in Haramaya District, Eastern Ethiopia, through simple random sampling. Data Research Topic entailed structured interviews, with subsequent entry into Epi Data version 3.1 for meticulous analysis utilizing SPSS version 21 software. Descriptive statistics summarized participant characteristics, while both bivariable and multivariable logistic regression analyses were conducted to elucidate factors associated with nutritional knowledge, setting statistical significance at *p*-value <0.05.

**Results:**

All 417 selected students participated in the study, yielding a commendable response rate of 100%. The median nutritional knowledge score among students stood at 58, with an interquartile range spanning from 44 to 66. Approximately 46.76% (95% CI: 42–51.59) of students exhibited good nutritional knowledge. Significant determinants of nutrition knowledge included sex [adjusted odds ratio (AOR) = 1.77, 95% CI: 1.03–3.04], being senior secondary students (AOR = 3.3, 95% CI: 1.95–5.73), and access to nutrition information (AOR = 3.3, 95% CI: 1.60–6.87).

**Conclusion:**

Our findings illuminate a notable level of nutritional knowledge among secondary school students in Haramaya District. However, discernible disparities in nutrition knowledge emerged based on gender, educational level, and access to nutrition information. These insights underscore the exigency of targeted interventions aimed at enhancing nutrition literacy among students, thereby fostering holistic health promotion endeavors.

## Introduction

In contemporary society, the significance of accurate nutritional knowledge, particularly among adolescents, cannot be overstated. It serves as a fundamental pillar in guiding their dietary choices toward those that promote optimal health and well-being (1). Nutritional literacy acts as a proactive measure against various health complications stemming from misinformation or ignorance concerning dietary practices (2).

The pivotal role of nutritional knowledge in addressing malnutrition is underscored by its potential to influence dietary habits positively. Schools, in particular, represent vital channels for imparting knowledge regarding healthy eating habits and lifestyles, given their accessibility to a significant proportion of the population aged between 4 to 18 years (3). This knowledge encompasses a broad understanding of dietary patterns, the interplay between diet and health outcomes, as well as culinary skills pertinent to food preparation (4).

Adolescents who engage in unhealthy dietary practices face a spectrum of health challenges including eating disorders, obesity, under nutrition, dental issues, and iron deficiency (5). Studies consistently demonstrate a positive correlation between heightened nutritional knowledge and healthier dietary behaviors, underscoring the critical need for comprehensive nutrition education (6).

Furthermore, a deficiency in nutritional knowledge has been associated with the global epidemic of obesity and overweight, both significant contributors to non-communicable diseases (7). Developing nations, like Ethiopia, confront substantial obstacles concerning malnutrition and non-communicable diseases, necessitating a concerted effort toward enhancing nutritional literacy and fostering healthy eating habits among adolescents (8). Ethiopia, in recent years, has witnessed a notable burden of malnutrition, overweight, and obesity, particularly within urban locales, largely attributed to a deficiency in nutritional knowledge (9).The perpetuation of inadequate dietary habits among adolescents can often be traced back to insufficient nutritional knowledge acquired during their formative years, predominantly influenced by familial and environmental factors (10).

Numerous studies conducted in various countries have explored the level of nutritional knowledge among adolescents. For instance, a study conducted in Kuwait revealed that enhancing nutritional knowledge could have a positive impact on the quality of food intake among adolescents (11). Similarly, research conducted in the Jimma zone of Ethiopia demonstrated that school-based health and nutrition education significantly contributed to the improvement of dietary practices (12). However, studies conducted in Lagos and Sokoto, Nigeria, indicated that overall nutritional knowledge among adolescents was poor, with only a small percentage having a good level of knowledge (13).

In Nigeria, a cross-sectional study found that a majority of respondents had poor knowledge and attitude toward reducing malnutrition, along with inadequate practices in this regard (2). Tanzania also reported similar results, with only a small percentage of adolescents demonstrating knowledge about the importance of nutritious food and its impact on academic performance (14). Meanwhile, in Kenya, a study found that the majority of adolescents had insufficient nutrition knowledge (15).

Another study conducted in Tanzania revealed that secondary school pupils who had nutrition subjects in their curriculum exhibited higher levels of knowledge compared to undergraduate students (14). Lastly, a study in Croatia found that knowledge about energy requirements and recommended servings of fruits and vegetables was lacking among adolescents (16). These studies collectively emphasize the necessity for effective nutrition education programs to enhance the nutritional knowledge of adolescents worldwide.

Moreover, the ramifications of inadequate nutritional knowledge extend into adulthood, potentially fueling the prevalence of lifestyle diseases associated with obesity (17). Conversely, a robust understanding of nutrition can positively shape dietary patterns, thereby mitigating the risk of non-communicable diseases and interrupting the cycle of intergenerational malnutrition (18). It’s imperative to note the scarcity of studies investigating the nexus between nutrition knowledge and dietary practices in Eastern Africa, particularly in Ethiopia. Existing research predominantly focuses on maternal nutritional knowledge and child nutrition, leaving a notable gap in understanding adolescent nutritional literacy, especially within the Haramaya District (19).

The forthcoming study aims to address this gap by assessing the nutritional knowledge and associated factors among secondary school students in the Haramaya District, Oromia region, Eastern Ethiopia. This endeavor not only seeks to shed light on the current status of nutritional literacy among adolescents but also holds promise in informing targeted intervention strategies by relevant governmental bodies and shaping future research trajectories in the domain of adolescent nutrition.

Furthermore, the study findings are poised to offer actionable insights for policymakers at various administrative levels, ranging from regional health bureaus to federal health ministries, facilitating the integration of nutritional programs into broader public health agendas. Ultimately, such initiatives hold the potential to foster improved adolescent health outcomes and alleviate the burden of non-communicable diseases on both individual and societal levels.

## Materials and methods

### Study area and period

The study was conducted at Haramaya District, situated in the East Hararghe zone of Eastern Ethiopia. Data research topic occurred between October 11 and October 31, 2022. Haramaya District is approximately 500 kilometers away from Addis Ababa, the capital city of Ethiopia, and encompasses 33 rural and two urban kebeles. There are five high schools in the District, which are: Haramaya Secondary School, Bate Secondary School, Adele Secondary School, Uggaz Lencha Secondary School, and Aweday Secondary School. The total number of students in five secondary schools were 12,392.

### Study design

An institution-based cross-sectional study design was employed.

### Population

All students in the randomly selected secondary schools within Haramaya District were study Population. But students in evening secondary schools and critically ill students were excluded from the study.

### Sample size determination and sampling techniques

The sample size was calculated using Epi-info population proportion formula considering a 55.8% prevalence of adequate nutritional knowledge from the previous study (20) with 0.05 level of significance, 0.05 margin error 95% confidence and adding 10% non-response rate as follows:


$$n=\frac{{\left(z\alpha /2\right)}^{2*}\left(P\right)*\left(1-p\right)}{d^{2}}$$

Where: n = minimum sample size required for study, p = estimated prevalence from previous study, Z _α/2_ = critical value at 95% confidence level of certainty, d = margin of error 5% and n = is sample size. Adding 10% of non-response rate the final sample size was 417.

**Figure 1:**
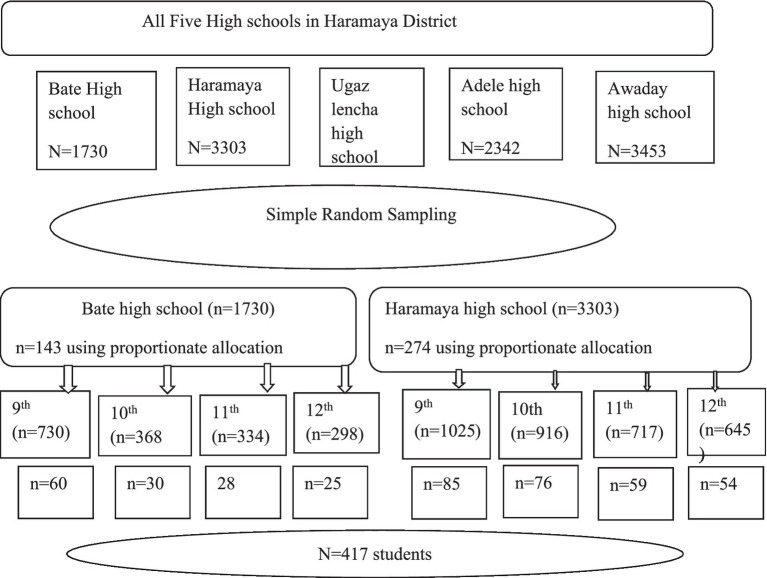
Sampling presentation of the study on nutritional knowledge and factors associated among secondary school students in Haramaya District, Oromia region, Eastern Ethiopia, 2023.

Two high schools were selected randomly selected from the total five high schools in the District using simple random sampling technique. Sampling frame was prepared for each grade (9th–12th) in the selected two high schools. From this list, participants were selected proportionally using simple random sampling technique from each secondary school (Figure 1).

### Data collection methods and tools

A structured questionnaire covering socio-demographic characteristics, lifestyle and behavioral factors, and nutritional knowledge was utilized. The questionnaire was self-administered in both English and Afaan Oromo languages. Data research topic was carried out by four diploma Nurses, under the supervision of two BSc Nurses. The questionnaire was pretested and adapted to include local foods.

Nutritional knowledge about seventeen (17) food items was evaluated to determine whether students had adequate knowledge about the nutrients, advantages of a diverse diet, and causes of malnutrition. A total nutrition knowledge score was obtained by summing the responses and assigning one point for each correct answer and zero otherwise. Responses that were correct were given a score of one, and those that were incorrect were given a score of zero. Responses with a value above the mean were considered good or adequate, while those below the mean were considered poor (20).

### Study variables

**Dependent Variable:** Nutritional knowledge.

**Independent Variables:**

***S*ocio-demographic characteristics**: **-** (Sex, Educational status of students, Residence, Mothers’ education, Fathers education, Mothers occupation, Fathers occupation).

**Life style and behavioral factors: -** (Access to drinking water, Source of water, Type of fuel used for cooking, Food preparation at home, Source of nutrition information, Frequency physical activity, Alcohol consumption Smoking).

### Data quality control

Data collectors and supervisors received training, pre-testing of questionnaires was conducted, and daily supervision ensured completeness and consistency of data. Double data entry and consistency checks were performed to maintain data quality.

### Data processing and analysis

Collected data was entered using Epidata Version 3.1 and analyzed using SPSS version 21 software. Descriptive statistics were conducted to describe the study participants. Bivariable logistic regression analysis was done to see the association between the dependent variable and each independent variables. The multicolinearity effect was checked by the variance inflation factor (VIF) and non-collinear covariates was included in the independent final binary logistic regression model to assess the possible association of outcome variables. All covariates that are significant at *p*-value <0.25 in the bivariable analysis was considered for multivariable analysis to control all possible confounders. Odd ratios along with 95% interval was estimated to identify factors associated with the outcome variable using multivariable logistic regression. The statistical significance was declared at *p*-value less than 0.05.

## Results

### Characteristics of participants

A total of 417 students participated in the study, yielding a response rate of 100%. Among the participants, the majority were senior secondary school students (55.9%) and male (65.7%). Approximately half 222 (53.2%) of the respondents hailed from rural areas. Concerning parental education, a notable proportion 115 (27.6%) of the mothers and 137 (32.9%) of fathers had attained only primary education, while a significant 124 (29.7%) number of mothers were involved in merchant activities, and fathers predominantly 171 (41.0%) working as farmers (Table 1).

**Table 1:** Socio-demographic characteristics of study participants at Haramaya District, Oromia region, Eastern Ethiopia (*n* = 417).

Characteristics	Frequency	Percentage
**Sex**		
Male	274	65.7
Female	143	34.3
**Educational status of students**		
Junior secondary school (grade9-10)	184	44.1
Senior secondary school (grade11-12)	233	55.9
**Residence**		
Urban	195	46.8
Rural	222	53.2
**Mothers education**		
No formal education	211	50.6
Primary	115	27.6
Secondary	67	16.1
College and above	24	5.8
**Fathers education**		
No formal education	81	19.4
Primary	137	32.9
Secondary	152	36.5
College and above	47	11.3
**Mothers occupation**		
Merchant	124	29.7
Government employee	87	20.9
Housewife	110	26.4
Other	96	23.0
**Fathers occupation**		
Farmer	171	41.0
Merchant	113	27.1
Government employee	60	14.4
Daily laborer	20	4.8
Other	53	12.70

### Lifestyle and behavioral factors

In terms of lifestyle-related behavior, most 310 (74.3%) of participants had access to water with a significant portion 68 (21.9%) utilizing tap water. Surprisingly, a considerable number 150 (36.0%) of participants used firewood for cooking. A majority 287 (68.8%) had received nutritional education, often from teachers 98(33.9%). Alcohol consumption were reported among the 149 (35.7%) participants (Table 2).

**Table 2:** Life style and behavioral factors of study participants at Haramaya District, Oromia region, Eastern Ethiopia (*n* = 417).

Characteristics	Frequency	Percentage
**Have access to drinking water**		
Yes	310	74.3
No	107	25.7
**Source of water (*n* = 310)**		
Borehole	152	49.0
Tap water	68	21.9
Dam/river	61	19.7
Other	29	9.4
Type of fuel used for cooking		
Electricity	168	40.3
Gas	64	15.3
Paraffin	30	7.2
Firewood	150	36.0
Other	5	1.2
**Food preparation at home**		
Mother	211	50.6
Father	93	22.3
Sister	61	14.6
Brother	34	8.2
Myself	18	4.3
**Nutrition information**		
Yes	289	69.30
No	128	30.70
**Source of information (*n* = 289)**		
Radio	71	24.6
Television/FM	90	31.1
Books	12	4.2
Teacher	98	33.9
Other	18	6.2
**Frequency physical activity**		
No	135	32.4
≤ 2 days/week	174	41.7
≥ 3 days/week	34	8.2
4 days/week	27	6.5
5 days/week	21	5.0
6 days/week	11	2.6
7 days/week	15	3.6
**Alcohol consumption**		
Never	205	49.2
Rarely	149	35.7
Daily/weekly	33	7.9
Monthly	30	7.2
**Smoking**		
Yes	155	37.2
No	262	62.8

### Nutrition knowledge and associated factors

The overall nutrition knowledge among the 417 students was 46.76%, with a median score of 58 and an interquartile range of 44 to 66. While the percentage of females with good nutritional knowledge was slightly higher than males, there was a significant increase in nutrition knowledge among senior secondary school students compared to junior secondary school students.

In the multivariable analysis, factors significantly associated with nutritional knowledge were identified. Female students were about 1.77 times more likely to have good nutritional knowledge compared to male students (AOR = 1.77, 95% CI: 1.03, 3.04, *p* = 0.039). Similarly, senior secondary school students were 3.3 times more likely to have good nutritional knowledge compared to junior secondary school students (AOR = 3.3, 95% CI: 1.95, 5.73, *p* < 0.001). Moreover, students who received nutrition information primarily from their teachers were 3.3 times more likely to have good nutritional knowledge compared to their counterparts (AOR = 3.3, 95% CI: 1.60, 6.87, *p* = 0.001) (Table 3).

**Table 3:** Multivariable logistic regression analysis for Factors associated with nutrition knowledge among secondary school students at Haramaya District, Oromia region, Eastern Ethiopia (*n* = 417).

		Nutrition knowledge			
Variable	Category	Good (*N*, %)	Poor (*N*, %)	COR 95% CI	AOR 95% CI
Sex	Male	121 (44.16)	153 (55.84)	1	1
	Female	74 (51.75)	69 (48.25)	1.36 (0.90, 2.03)	1.77 (1.03,3.04)*
Students’ education	Junior secondary	60 (32.61)	124 (67.39)	1	1
	Senior secondary	135 (57.94)	98 (42.06)	2.85 (1.90, 4.26)***	3.3 (1.95, 5.73)***
Fathers education	No formal education	31 (38.27)	50 (61.73)	1	1
	Primary	65 (47.45)	72 (52.55)	1.45 (0.83, 2.55)	0.96 (0.40, 2.14)
	Secondary	74 (48.68)	78 (51.32)	1.53 (0.88, 2.65)	1.11 (0.51, 2.43)
	College and above	25 (53.19)	22 (46.81)	1.83 (0.86, 3.793)	0.983 (0.33, 2.90)
Fathers occupation	Farmer	74 (43.27)	97 (56.73)	0.67 (0.37, 1.20)	0.84 (0.34, 2.09)
	Merchant	49 (43.36)	64 (56.64)	0.67 (0.36, 1.26)	0.57 (0.22, 1.43)
	Gov’t employee	32 (53.33)	28 (46.67)	1	1
	Non-gov’t employee	10 (50)	10 (50)	0.87 (0.32, 2.41)	0.43 (0.99, 1.89)
	Daily laborer	23 (43.40)	30 (56.60)	1.14 (0.54, 2.40)	0.50 (0.17, 1.45)
Food preparation	Mother	93 (44.08)	118 (55.92)	1	1
	Father	41 (44.09)	52 (55.91)	1.0 (0.61, 1.63)	1.83 (0.84, 3.99)
	Sister	35 (57.38)	26 (42.62)	1.7 (0.96, 3.04)	1.56 (0.75, 3.27)
	Brother	19 (55.88)	15 (44.12)	1.6 (0.77, 3.33)	1.75 (0.67, 4.55)
	Myself	7 (38.89)	11 (61.11)	0.80 (0.30, 2.16)	0.40 (0.13, 1.25)
Source of information (*n* = 289)	Radio	34 (47.89)	37 (52.11)	1.20 (0.64, 2.24)	1.55 (0.76, 3.16)
	Television	39 (43.33)	51 (56.67)	1	1
	Books	3 (25.00)	9 (75.00)	0.44 (0.11, 1.72)	0.79 (0.18, 3.56)
	Teacher	55 (56.12)	43 (43.88)	1.67 (0.94, 2.98)	3.3 (1.60, 6.87)**
	Other	7 (38.89)	11 (61.11)	0.83 (0.29, 2.34)	1.20 (0.37, 3.85)

AOR, adjusted odds ratio; COR, crude odds ratio; CI, confidence interval; *p-value < 0.05; **p-value < 0.01.

## Discussion

Nutrition knowledge stands as a cornerstone in fostering healthier dietary habits among individuals, empowering them to make informed choices despite external influences such as advertising. This study sought to evaluate the level of nutrition knowledge and its determinants among secondary school students in Haramaya District, Oromia region, Eastern Ethiopia.

The overall nutrition knowledge among students was assessed at 46.76%, with a median score of 58 and an interquartile range of 44 to 66. When compared with analogous studies, these findings are consistent with research conducted in Mississippi (21), Iran (22) and in China (23). But lower than studies in Egypt (24), Indonesia (25), New Zealand (26), Iran (27), and Turkey (28). Conversely, this is higher than studies in Kenya (15), Iran (29). Such variations may be attributed to discrepancies in educational curriculum, study settings, sample sizes, and socio-economic factors.

This study delved into demographic characteristics and factors influencing nutrition knowledge. It was observed that female students were roughly twice as likely to possess nutritional knowledge compared to their male counterparts. This observation resonates with findings from Romania, Michigan, and Turkey (30–32), potentially reflecting females’ heightened concern regarding body image, weight, and dietary habits.

In addition, females seek nutritional counselling from professionals more frequently than males (33). Furthermore, female students reported more often than males that they cooked on a daily basis (always) and prepared their own meals is could be another possible explanation for the higher knowledge score achieved by females (34).

Furthermore, senior secondary students exhibited a threefold likelihood of possessing nutritional knowledge compared to their counterparts at lower educational levels. The previous study reported that secondary education could positively associated with nutritional knowledge than primary school children (35). Likewise, in 2012 the study conducted in Turkey acknowledged that teaching nutrition and providing educational materials can induce positive changes in nutritional knowledge (36). This suggests a positive impact of educational interventions on nutritional literacy, as corroborated by previous research.

The current study also demonstrated that the odds of nutritional knowledge was 3 times higher among students who were received nutrition information primarily from their teachers than their counterparts. This finding is consistent with findings of a previous study conducted in Iran and Taiwan (37, 38). The possible reason might be Teachers’ knowledge and food-related beliefs and behaviors can influence the habits of their student to adopt the healthy eating practices as teachers themselves could be role model for his students (39, 40).

Additionally, students who received nutrition information from their teachers were thrice as likely to exhibit good nutritional knowledge. This underscores the pivotal role of educators as both role models and facilitators in fostering healthy eating habits among students.

### Strengths and limitations

The utilization of a standardized and pretested checklist facilitated comparisons with national and international standards. The study provided valuable insights into the proportion of nutritional knowledge within the study area. The cross-sectional study design inherently restricts the establishment of cause-effect relationships. The facility-based nature of the study may limit its generalizability to the broader population within the catchment area.

## Conclusion

The study unveiled substantial nutritional knowledge among high school students. There exists a compelling need for robust nutrition education interventions to enhance dietary decision-making, given the significant impact of adolescent behavioral patterns on long-term health outcomes.

Develop and implement a comprehensive nutrition education program tailored for high school students, leveraging educational institutions as pivotal platforms for disseminating nutritional knowledge. Harness school-based nutrition education initiatives to reinforce the importance of nutritional literacy among students. Future research endeavors should contemplate integrating factors such as wealth index and cultural influences into the assessment of nutritional knowledge.

## Data availability statement

The original contributions presented in the study are included in the article, further inquiries can be directed to the corresponding author/s.

## Ethics statement

Ethical clearance was obtained from the institutional health research ethics review committee (IHRERC) of CHMS the Haramaya University granted approval for this study under reference number C/AC/R/D/01/3063/22 on 04/08/2022. A written permission letter was written from Haramaya University and submitted to Haramaya District Education office. For all directors’ and participants’ information was given about the study before the data collection on its possible risk, benefit, confidentiality, privacy, its voluntary activity, right of with-drawl, and the time the questionnaire was taken. After explaining the objective of the research to participants, informed consent with a written signature was acquired. They were informed that they could withdraw at any time and/or refrain from answering questions. Participants in the study were also notified that all data obtained from them would be kept confidential by using code rather than any personal identifiers. Furthermore, the research procedure was carried out in compliance with the Helsinki Declaration of the World Medical Association.

## Author contributions

AE: Conceptualization, Data curation, Formal analysis, Investigation, Methodology, Project administration, Resources, Software, Validation, Visualization, Writing – original draft, Writing – review & editing. KJ: Conceptualization, Data curation, Formal analysis, Investigation, Methodology, Project administration, Resources, Software, Supervision, Validation, Visualization, Writing – original draft, Writing – review & editing. IA: Conceptualization, Data curation, Formal analysis, Investigation, Methodology, Resources, Software, Validation, Visualization, Writing – original draft, Writing – review & editing. BG: Conceptualization, Data curation, Formal analysis, Investigation, Methodology, Project administration, Resources, Software, Supervision, Validation, Visualization, Writing – original draft, Writing – review & editing. AB: Conceptualization, Data curation, Formal analysis, Investigation, Methodology, Software, Validation, Visualization, Writing – original draft, Writing – review & editing. MD: Conceptualization, Data curation, Formal analysis, Investigation, Methodology, Software, Supervision, Validation, Visualization, Writing – original draft, Writing – review & editing. MA: Conceptualization, Data curation, Formal analysis, Investigation, Methodology, Software, Validation, Visualization, Writing – original draft, Writing – review & editing.
